# Same pattern, different mechanism: Locking onto the role of key species in seafloor ecosystem process

**DOI:** 10.1038/srep26678

**Published:** 2016-05-27

**Authors:** Sarah Ann Woodin, Nils Volkenborn, Conrad A. Pilditch, Andrew M. Lohrer, David S. Wethey, Judi E. Hewitt, Simon F. Thrush

**Affiliations:** 1 Department of Biological Sciences, University of South Carolina, 701 Sumter Street, Columbia, South Carolina 29208, USA; 2 School of Marine and Atmospheric Sciences, Stony Brook University, Stony Brook, New York 11794-5000, USA; 3 School of Science, Faculty of Science and Engineering, University of Waikato, Hamilton 3240, New Zealand; 4 National Institute of Water and Atmospheric Research, PO Box 11-115, Hamilton 3251, New Zealand; 5 Institute of Marine Science, University of Auckland, Private Bag 92091, Auckland, 1142, New Zealand

## Abstract

Seafloor biodiversity is a key mediator of ecosystem functioning, but its role is often excluded from global budgets or simplified to black boxes in models. New techniques allow quantification of the behavior of animals living below the sediment surface and assessment of the ecosystem consequences of complex interactions, yielding a better understanding of the role of seafloor animals in affecting key processes like primary productivity. Combining predictions based on natural history, behavior of key benthic species and environmental context allow assessment of differences in functioning and process, even when the measured ecosystem property in different systems is similar. Data from three sedimentary systems in New Zealand illustrate this. Analysis of the behaviors of the infaunal ecosystem engineers in each system revealed three very different mechanisms driving ecosystem function: density and excretion, sediment turnover and surface rugosity, and hydraulic activities and porewater bioadvection. Integrative metrics of ecosystem function in some cases differentiate among the systems (gross primary production) and in others do not (photosynthetic efficiency). Analyses based on behaviors and activities revealed important ecosystem functional differences and can dramatically improve our ability to model the impact of stressors on ecosystem and global processes.

Humanity’s influences on ecosystems have broad implications for the Earth’s future. Models of global biogeochemical cycles make significant contributions to predicting future scenarios, but just as the human species has influenced change, we need to address the role of other species to ensure our understanding of rates and processes is sufficient to provide good estimates of state and trends. On an aerial basis the seafloor is the largest ecosystem on earth, has some of the steepest known chemical gradients, and can be extraordinarily productive and reactive, particularly in shallow water^1^,^2^,^3^. The biodiversity crisis, climate change and recognition of marine ecosystem services have brought into focus the role of seafloor ecosystems in many global processes^4^,^5^. However, our broad-scale assessments appear to be missing important details or mechanistic understanding, as detailed below. This missing process-based knowledge could fundamentally affect both our predictions of status and trends in marine ecosystems, and also constrain our ability to affect positive change. Global nitrogen budgets, for example, do not balance; seafloor sediments are implicated because of their role both supporting primary production and releasing nitrogen gases to the atmosphere by denitrification^6^,^7^. The ecosystem processes that underpin this cycle are interconnected and, particularly in coastal seas, involve strong links between the water column and the seafloor^8^,^9^.

Surface layers of seafloor sediments, where animals live, are highly heterogeneous, with ecosystem engineering organisms directly generating physical habitat structure^10^,^11^,^12^,^13^,^14^ and influencing hydrodynamic and biogeochemical processes^7^,^15^,^16^,^17^. The activities of large and active species drive many integrated measures of ecosystem process and function such as bioturbation, nutrient release from sediments, and benthic primary production^12^,^18^,^19^,^20^,^21^. Important animal activities include sediment movement and bioirrigation that alter rates of inorganic nitrogen release from sediments, and thus are important both in terms of non-local transport driving benthic-pelagic coupling and microphytobenthic primary production^17^,^21^,^22^. Until recently our understanding of these processes has been limited by our inability to observe in real time the behavior of animals buried in the sediment. Now a combination of porewater pressure measurements, observations of changes in sediment surface topography, and time-resolved imaging of oxygen flux through the sediment column have provided new perspectives as well as enhanced quantification of impacts of behaviors^15^,^23^,^24^,^25^,^26^.

Contributions to global budgets from the seafloor will be driven by how ecosystem engineers drive the flux of particles and solutes, influencing the spatial and temporal dynamics of processes within the sediments. Organisms can physiologically influence flux across the seafloor through respiratory and excretory processes. But potentially more critical are the effects of ecosystem engineering organisms on sediment biogeochemistry. A key controller here is the behavior of the animals, their size and abundance, what type of sediments they live in, and where they live relative to strong chemical gradients, particularly oxygen, in the sediment. Physical advection of nutrient rich porewater to the overlying water associated with waves and benthic boundary flow is important, but a recent synthesis emphasizes biological over physical processes in sediments with permeabilities <10^−12^ m^2 ^27. Significant pressure gradients generated by macrofauna have been recorded in permeabilities from 9.9 × 10^−14^ m^2^ to 5.9 × 10^−11^ m^2 ^14. Thus, in sands, both physical and biotic porewater advection can be important. By driving overlying water deep into sediment, displacing ammonium, dissolved inorganic carbon and silicate rich porewaters without mobilizing sediment particles, the hydraulic behavior of animals can enhance primary productivity far beyond the effects of macrofaunal excretion^16^,^17^,^21^,^22^,^28^,^29^.

We contend that the processes at the meter scale matter because organisms and their behavior influence ecosystem function. A critical ecological question is which aspects of environment, natural history, and behavior drive the delivery of function(s). For example for nutrient fluxes, is it sediment turnover by bulldozing organisms with associated disruption of the porewater and changes in surface topography^30^ or direct porewater pressurization by advective bioirrigation with little or no sediment grain movement^26^ or is it just the excretion rates of very large and numerous organisms? The spatial dynamics associated with specific behaviors are very different; bulldozing directly causes grain and porewater movements near to the animal’s body and alters surface topography, while pressurization of porewater can cause water movement several body lengths beyond the pressure source^15^,^23^,^26^. Temporal dynamics are affected as well because infauna move relatively slowly through the sediment; so, tracks are created over hours or days^31^. In contrast, hydraulic forces associated with feeding or respiration are more frequent with temporal scales of seconds to minutes^15^,^23^,^24^,^26^. If we are to scale up from point measurements on the seafloor or scale-down in broad-scale ecosystem models, then defining these processes and their links to mechanisms is critical. Models built without links to mechanism are likely to fail, especially as the assumption of stationarity becomes more problematic with accelerating environmental change^32^,^33^.

Here we show how advective bioirrigation and particle reworking are modulated by three very different ecosystem engineers: *Macomona liliana* (porewater-pressurizing, deposit feeding bivalve); *Austrovenus stutchburyi* (clump-forming, shallow burying, suspension feeding bivalve); and *Echinocardium cordatum* (bulldozing, deposit feeding urchin) (Supplementary Table S1). Our fundamental thesis is that based on knowledge of the natural history, animal behavior, and sediment characteristics, all of which are fine scale species driven metrics, we can predict the drivers of ecosystem function in systems dominated by one of the above ecosystem engineer species. New techniques, such as porewater pressure sensors, allow us to quantify the impacts of fine scale, species specific behaviors in ways that generate predictions of magnitudes of such important integrated components of ecosystem function as ammonium efflux. We can then test our predictions with published field measurements in systems dominated by each of the three ecosystem engineers.

Two generalizable predictions concerning ecosystem function but based on knowledge of species specific behaviors are as follows: (1) ammonium efflux from the sediment in the absence of primary production, and adjusted for excretion by large macrofauna, will be greater in sediments dominated by organisms driving deep bioadvection than in sediments with only shallow burrowers or in sediments dominated by axial bulldozers that push sediments laterally; (2) a xial bulldozing generates topography at the sediment-water interface, mixes porewaters, and increases diffusion, dependent on bulldozing rate, burial of fresh organic material, and the degree of physical sediment transport.

These two general predictions have the following implications for the ways our three ecosystem engineers will affect ecosystem processes (for additional details see Supplementary Table S2). (1) Benthic primary production will be enhanced by the excretion of *Austrovenus* and thus be a positive function of their size and density^34^,^35^,^36^. (2) Enhancement of benthic primary production by *Macomona* will be primarily due to their hydraulic activities, not excretion, but again positively dependent on size and density^26^. (3) Enhancement of benthic primary production by *Echinocardium* will be primarily a function of degree of rugosity of the sediment surface and bulldozing rate and thus a positive function of size and density^30^. (4) Benthic primary production scaled to chlorophyll *a* concentration will be approximately equal across all three ecosystem types reflecting equivalency in efficiency, implying absence of nutrient limitation in beds of any of these species but dependent on very different mechanisms of nutrient delivery: excretion (*Austrovenus*), bioadvective forcing of porewater (*Macomona*), and bulldozing (*Echinocardium*). Herein we predict ammonium efflux, an integrated component of ecosystem function, from quantitative knowledge of activity differences and then test those laboratory derived values against field measurements previously published by several of the authors (see Supplementary Table S3).

## Results

### Behavior and porewater flux

**Figure 1:**
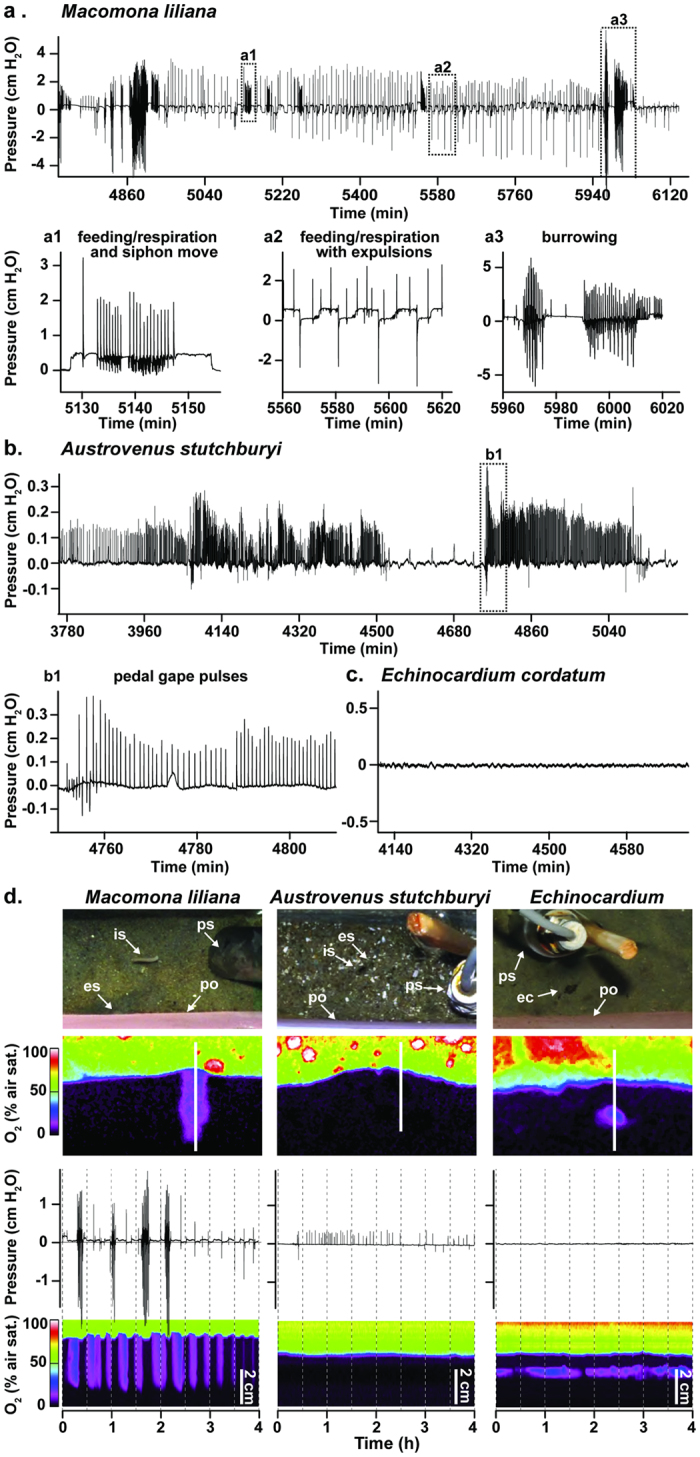
Behaviorally generated porewater pressure dynamics of *Macomona*, *Austrovenus*, and *Echinocardium* and links to oxygen dynamics. Parts (**a–c)** ordinate in cm of water pressure, time in minutes on the abscissa. Part (**a**) *Macomona* exhibit complex behaviors with specific wave forms: exhalent siphon movement is typically accompanied by a series of large, primarily positive pressure pulses (a1); during surface deposit feeding the water exits the exhalent siphon within the sediment resulting in small but sharp pressure rises above hydrostatic baseline, accompanied by occasional sharp positive pressure pulses (labial palp cleanouts) and negative pressure pulses (pseudofeces expulsions and defecations) (a2); burrowing is seen as large positive and negative pulses (a3). Part (**b**) *Austrovenus* exhibits weak effects on porewater pressures: burrowing results in positive and negative pulses similar to those of *Macomona* but much smaller; feeding is accompanied by sharp, small positive pulses that often result from closure of the excurrent siphon and apparent opening of the pedal gape, perhaps for removal of material from the labial palps^38^ (b1). Part (**c**) *Echinocardium* has no detectable effects on porewater pressures. Part (**d**). A continuous four hour record of oxygen dynamics and synchronized pressure dynamics for *Macomona, Austrovenus*, and *Echinocardium*. Top panel: surface of sediment with organism and pressure sensor (‘is’ inhalant siphon; ‘es’ exhalent siphon; ‘ps’ pressure sensor; ‘po’ planar optode; ‘ec’ aboral surface of *Echinocardium*). Second panel: planar optode image of sediment oxygen concentration (bright colors indicate presence of oxygen, see scale lower left). Sizes of optodes shown (entire optode size): *Macomona*: 8.6 × 5.2 cm (20 × 20 cm), *Austrovenus*: 10.6 × 6.3 cm (20 × 13 cm), *Echinocardium*: 10.1 × 6.0 (20 × 13 cm). Third panel: pressure signals in cm of water pressure, time in hours. Fourth panel: oxygen dynamics along vertical spatial gradient into the sediment (see white bar in second panel) as revealed by the changes in luminescence-lifetime of the optode with times synchronized to those of the pressure record in panel three.

A major contribution to our results stems from our observations of animal behaviors and their effects on porewater flux (Fig. 1). Common behaviors of *Macomona liliana* were burrowing, siphon relocation, defecation/pseudofecal expulsion, and feeding/respiration^26^,^37^ (Fig. 1a,a1–a3). The feeding and respiratory activities of *Macomona* lead to pressurization of the sediments ~50% of the time and are clearly linked to oxygenation of the subsurface sediment and advective transport of porewater through the sediment water interface (Fig. 1a,d: panels 3 and 4; Table 1). The apparent changes in position of the oxic-anoxic boundary seen in the optode with *Macomona* are due to expulsion of low oxygen porewater; no change in sediment height occurred (Fig. 1d: bottom panel). For *Austrovenus stutchburyi* only two types of hydraulic behavior were common (Table 1). Sharp positive pulses were the most common and were synchronous with closure of the excurrent siphon (Fig. 1, b1)^38^. Additionally, there were less frequent, sharp negative pulses due to fecal and pseudofecal expulsions that were associated with valve claps. Little evidence of oxygenation of the subsurface sediment or expulsion of porewater into the overlying water was seen on the optode (Fig. 1d). *Echinocardium cordatum* had no measurable hydraulic activity, with the optode only showing the oxic halo around individuals (Fig. 1d); no detectable changes in porewater pressures were recorded (Fig. 1c,d).

### Ammonium efflux

Ammonium effluxes measured in *Echinocardium* assemblages (Table 2: mean 10.1, range 2.3 to 21.4 μmol m^−2^ h^−1^) were consistent with our prediction, which was based on the assumption that bulldozing by *Echinocardium* is causing replacement of all of the porewater contained within the displaced sediments (expected value 10 μmol m^−2^ h^−1^) (Table 3). We estimated bioadvective ammonium efflux for the *Macomona* population studied as 289 μmol m^−2^ h^−1^ (Table 3). This is based on the time averaged pumping rates for small *Macomona* (2.35 cm length, 12.9 mL h^−1^ ind^−1^)^29^ and the measured integrated porewater ammonium concentration of 100 μmol L^−1^ from the *Macomona* site. This reflects the rate of advective bioirrigation by the population of 2.9 L m^−2^ h^−1^ (69.6 L m^−2^ d^−1^) in a sediment containing approximately 20 L m^−2^ porewater down to 5 cm depth.

**Figure 2:**
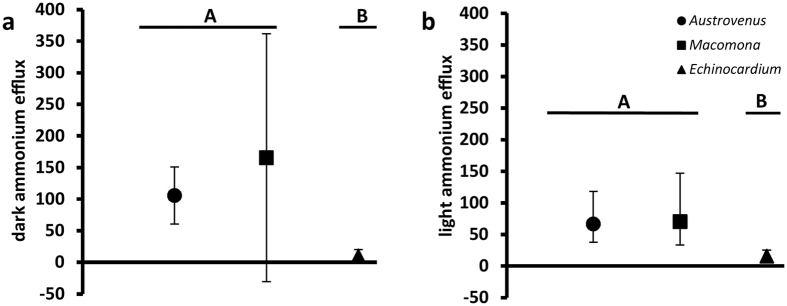
Parts (**a,b**) Ammonium efflux (μmol m^−2^ h^−1^) versus assemblage type ▪ ▴ Data sources: *Austrovenus*^35^; *Echinocardium*^46^,^52^; *Macomona*^53^. Means and standard deviations of untransformed data for ammonium release from the sediments in the dark (part **a**) and log_10_ back transformed data for release in the light (part **b**). The dark efflux data had non-homogeneous variances and were non-normal, not corrected by transformation; so, the non-parametric Kruskal-Wallis rank sum test was used for statistical evaluation. A log_10_ transform corrected non-normality of the light flux data. Dark flux: Kruskal-Wallis test statistic = 6.23, df = 2, p < 0.05; light flux: ANOVA on log_10_ transformed values: F_2,9_ = 5.74, p < 0.05. Solid horizontal lines separated by a space and labelled with different letters indicate statistical significance by Holm-Šidák a posteriori tests, adjusted p < 0.05: dark efflux: *Austrovenus:Macomona* p = 0.5, *Austrovenus:Echinocardium* p < 0.05, *Macomona:Echinocardium* p < 0.05; light efflux: *Austrovenus:Macomona* p = 0.92, *Austrovenus:Echinocardium* p < 0.05, *Macomona:Echinocardium* p < 0.05.

Ammonium release from the sediments (efflux) in the dark and in the light was significantly lower in the *Echinocardium* assemblage than in assemblages with either of the bivalves (Fig. 2). As predicted (Supplementary Table S2), the ammonium efflux in the dark for the *Austrovenus* assemblage was similar to expected rates of excretion (Table 2), whereas, for the *Macomona* assemblage, advective bioirrigation by the population appeared to be responsible, not excretion (Tables 2 and 3). In the *Echinocardium* assemblage, sediment turnover and enlarged surface area for diffusion probably were the source of ammonium efflux (Table 3) as suggested previously^30^,^39^. In the *Austrovenus* and *Macomona* assemblages, ammonium efflux in the dark and in the light was sufficiently large as to suggest enhancement of water-column productivity as well as benthic productivity. Again this was not true of the *Echinocardium* assemblage (Fig. 2, Table 2). Comparisons of the three species highlight the importance of the temporal and spatial dynamics of slow processes measured over hours to days (bulldozing) versus a fast process (bioadvection), driven by respiration and feeding activities and measured over seconds to minutes, and an intermediate process (excretion) measured over hours (Tables 1, 2, 3, Fig. 1)^26^.

### Benthic primary production

**Figure 3:**
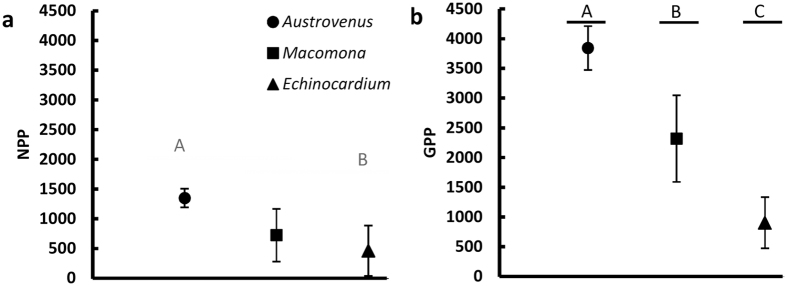
Net (NPP) (part (**a**) and gross primary productivity (GPP) (part (**b**) (mean ± SD) (μmol O_2_ m^−2^ h^−1^) measured in benthic flux chambers versus assemblage type. For data sources see Fig. 2. NPP: ANOVA: F_2,9_ = 4.12, p = 0.0537; Tukey a posteriori tests: *Austrovenus:Macomona* p > 0.1, *Austrovenus:Echinocardium* p = 0.053, *Macomona:Echinocardium* p > 0.63. GPP: ANOVA: F_2,9_ = 17.7, p < 0.001; Tukey a posteriori tests: *Austrovenus:Macomona* p < 0.025, *Austrovenus:Echinocardium* p < 0.001, *Macomona:Echinocardium* p < 0.025. Solid grey horizontal lines separated by a space and labelled with different letters indicate marginal significance by Tukey’s a posteriori tests. Solid black lines indicate statistical significance, adjusted p < 0.05.

**Figure 4:**
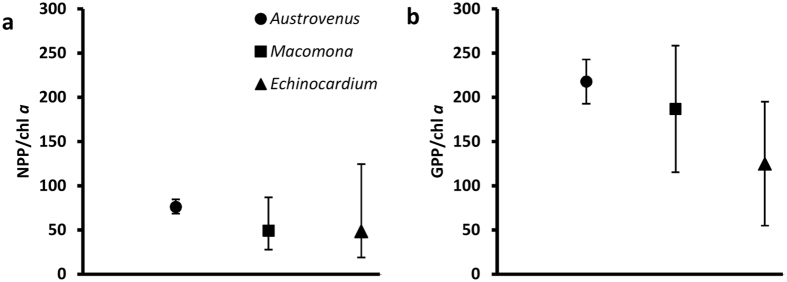
Net (NPP) (part (**a**) and gross primary productivity (GPP) (part (**b**) (μmol O_2_ m^−2^ h^−1^) normalized by sediment chlorophyll *a* content (μg g^−1^ dry weight of sediment) (mean ± SD) versus assemblage type. NPP/chl *a*: ANOVA on log_10_ transformed values: F_2,9_ = 0.578, p > 0.58; GPP/chl *a*: ANOVA on untransformed values: F_2,9_ = 1.67, p > 0.24.

Gross primary production m^−2^ (GPP) corresponded to the pattern of ammonium efflux and chlorophyll *a* concentration (Fig. 2, Supplementary Table S4) with production in the *Austrovenus* assemblages >those of *Macomona* >those of *Echinocardium* (Fig. 3b). A similar pattern was seen for net primary production m^−2^ (NPP) though only the NPP of the *Austrovenus* assemblage was even marginally significantly different from that of *Echinocardium* (Fig. 3a). To address the efficiency of primary production, measurements of NPP and GPP m^−2^ were normalized by sediment chlorophyll *a*. In none of the three assemblage types was photosynthetic efficiency different, suggesting no nutrient limitation even though ammonium efflux, our proxy for nutrient regeneration, was so much lower in the beds with *Echinocardium* (Figs 2 and 4). However, for both NPP and GPP normalized by sediment chlorophyll *a*, but particularly for GPP/chl *a*, there is an apparent, though non-significant, pattern in mean values (Fig. 4b). This inability to detect significant differences was related to the higher spatial variability in normalized productivity in *Macomona* and *Echinocardium* assemblages relative to *Austrovenus* assemblages. This is reflected in the pattern of coefficients of variation for photosynthetic efficiency (NPP/chl *a*: 0.09, 0.43, and 0.61; GPP/chl *a*: 0.11, 0.38, 0.56; *Austrovenus, Macomona, Echinocardium* respectively). Again this is suggestive of increased spatial and temporal variability in patterns driven by activities versus density/size alone.

## Discussion

Our analysis demonstrates that each species drives the systems in which they are abundant by very different mechanisms; yet, metrics which integrate the functioning of an ecosystem, such as photosynthetic efficiency, GPP m^−2^, and NPP m^−2^, are not equivalent in detecting differences. GPP m^−2^ reveals significant differences among all three assemblages, while NPP m^−2^ fails to distinguish the *Macomona* assemblage (Fig. 3). In contrast, photosynthetic efficiency cannot statistically separate any of the three assemblages (Fig. 4). The latter, however, dramatically highlights the greater inherent variance of the *Macomona* and *Echinocardium* assemblages, where patterns are driven by activities of the population (Fig. 4). Recognition of this variance and its link to activity strongly suggests that sublethal influences may be of greater magnitude in such assemblages as performance drops with increasing stress. In many ways, variance is the measure of the sometimes subtle differences among organisms that mechanisms reflect. Metrics with higher degrees of spatial and temporal averaging obscure such underlying mechanisms^40^. Failure to consider mechanism when estimating the impact of stressors on assemblages can easily lead to false predictions since it is the impact on the forcing functions and their interactions, not the resultant metrics, that are critical in making informed predictions about effects of stressors on ecosystems^33^,^41^.

Our understanding of the differences in mechanism represented by these three ecosystem engineers derives from quantification of their activities and of the consequences of those activities for primary production (Tables 1, 2, 3). The essential component is an understanding of the animal’s behaviors and how those behaviors are likely to interact with sediment biogeochemistry. Without that understanding we would not have known which activities to quantify nor would we have understood the reasons for the differences in variance among the assemblages.

The predicted ammonium efflux for the *Macomona* population was 289 μmol m^−2^ h^−1^ (Table 3). This exceeds the efflux measured in the dark, suggestive of a) nitrification occurring with the increased availability of oxygen due to bioadvection^42^,^43^,^44^; b) continued, but unexpected, uptake of ammonium in the dark by microphytobenthos; or c) reduced activity of *Macomona*. The measured efflux is comparable to measurements for the congener *Macoma balthica* (167 to 208 μmol m^−2^ h^−1^) where the occurrence of nitrification was also suggested^42^.

Ammonium efflux by the *Echinocardium* beds is low relative to the other two assemblages, both in the dark (10 to 16 fold difference) and in the light (4 to 5 fold difference) (Table 2, Fig. 2). The *Echinocardium* beds are, however, quite productive, especially on a per chlorophyll *a* basis, and none of the three bed types are statistically different from one another in NPP/chl *a* or GPP/chl *a* (Fig. 4), although variability increases from *Austrovenus* beds to *Echinocardium* beds. One possibility is that the high productivity and variability of the *Echinocardium* beds is due to the greater surface area of the rugose bulldozed surface compared to the undisturbed sediment surface. The tracks of *Echinocardium* are 3.4 cm wide and 3.8 cm deep^39^; so, the rugose surface of the *Echinocardium* bed has at minimum 30% more surface area than an undisturbed surface, thus greatly increasing the surface area available for photosynthesis and diffusion.

The ammonium effluxes for *Macomona* assemblages also have a much higher degree of variance than those for *Austrovenus* assemblages although the effluxes are not significantly different either in the light or the dark (Fig. 2). Given the source of the efflux, bioadvection by *Macomona* versus excretion of *Austrovenus*, this is likely to have different spatial effects. Organism size will influence ammonium excretion and activity rates^29^, but porewater pressure effects will be influenced by spatial variation in sediment tortuosity and permeability, the steepness of the nutrient gradients and the position of the individual’s excurrent siphon - with deeper excurrent ejections likely to drive higher ammonium effluxes^45^.

The activity of these animals can explain some counter intuitive results. *Macomona* behavior drives ammonium efflux allowing us to use efflux as a proxy for *Macomona* activity, specifically feeding. Ammonium efflux in *Macomona* dominated assemblages therefore should be inversely related to chlorophyll *a* standing stock; the more grazing, the less standing stock of primary producers is expected, which is indeed what we observe (a negative slope and positive intercept both in the light (slope: −0.03, intercept: 15.22, adjusted R^2^ 0.67) and in the dark (slope: −0.01, intercept: 14.13, adjusted R^2^ 0.62)).

In contrast, for *Echinocardium*, increases in sediment surface area associated with bulldozing activity will increase the area for photosynthesis, diffusion and advection, presumably driving the positive relationship between *Echinocardium* density and chlorophyll *a* content as well as primary production previously reported^30^,^31^,^46^. Ammonium efflux should not therefore be a good indicator of *Echinocardium* feeding, but rather of bulldozing activity and surface rugosity. Thus, rather than a negative relationship between ammonium efflux and standing crop of chlorophyll *a* as found for *Macomona*, we find a positive relationship (light: slope: 0.19, intercept: 4.29, adjusted R^2^ 0.91; dark: slope: 0.16, intercept: 5.84, adjusted R^2^ 0.97). For *Austrovenus* ammonium efflux does not reflect activity, but rather excretion. Neither in the light nor in the dark was there a significant relationship between ammonium efflux and standing crop of chlorophyll *a* (light: slope: 0.01, intercept: 16.61, adjusted R^2^ 0.40; dark: slope: −0.003, intercept: 17.95, adjusted R^2^ ~ 0).

The results obtained strongly suggest that these large ecosystem engineers drive these systems such that the measured values correlate with those estimated from their activities and abundances (Tables 1, 2, 3, Supplementary Table S2). An argument could be made that there are other important species such as micro-herbivores which are critically important^47^. Given that these ecosystem engineers are known to affect the composition of the community and the shape and strength of the interaction network^41^, it is quite likely that the components of the assemblages associated with each of these species are quite different and in fact this is known to be true^19^,^41^. The primary point, however, is that using the characteristics and behaviors of the three ecosystem engineers alone and their densities, the resulting predictions for properties such as ammonium efflux were consistent with the measured values from the field. Our ability to relate information on the actions of the three ecosystem engineers alone to our field measurements demonstrates that for the ecosystem functions we studied, these species were critically important. Their behaviors and densities drove the patterns predicted and tested successfully.

Comparative measurement of behavior and ecosystem function across habitats provides insight into whether summary metrics, such as net or gross primary productivity m^−2^ or photosynthetic efficiency, reflect the underlying network of processes. One tends to correlate response variables with the numerically or biomass dominant organism as the driver. With *Austrovenus* this is a valid choice given the link to excretion and the thousands of individuals per meter square. However, when the organism’s activities drive dynamics, the species is not necessarily the most abundant organism, although it still may be the biomass dominant^20^,^21^. This is the situation for both *Macomona* and *Echinocardium* assemblages. Abundances or biomass are important multipliers and modulators of activity rates commonly used in numerical models that specifically consider the role of biota in ecosystem processes. But as our results highlight, it is the activities (and rates of activities) of the organisms and how they interact with the environment that drive the system (Fig. 1; Tables 1, 2, 3; Supplementary Table S2).

Although many uncertainties exist in global models, continental shelves <50 m water depth, which only account for 2% of the area of the ocean, are very important in global flux of organic carbon to the seafloor and particulate organic carbon burial^48^. The upper layers of seafloor sediments are extremely reactive with more chemical processes than the overlying water column^49^. Ecosystem engineers of the types discussed here are very common in these important shallow water habitats. To date, it has been hard to reconcile the interactions of animal behaviors, biogeochemical gradients and primary production, but it is critical to do so. This is particularly true given the increasing evidence for impacts of ocean acidification and global warming on animal behavior^50^. Our predictions allowed us to reveal the importance of several drivers of benthic primary production in different benthic ecosystems: release from nutrient limitation by excretion (*Austrovenus*); release from nutrient limitation by bioadvection (*Macomona*); changes in the rugosity of the sediment surface and thus the surface area to volume relationship plus enhancement of physical advection and release of porewater (*Echinocardium*).

While it is recognized that sediment modelling is complex^51^, the range of behaviors and environmental contexts for different ecosystem engineers working on the seafloor makes prediction of their effects complicated. This level of complication has often resulted in seafloor processes being ignored in global budgets or the seafloor being represented as a benthic black box. A black box may seem appropriate for a global budget model if various combinations of process produce the same average net functional effect via very different mechanisms, but this will not give any clues as to the consequences of shifts in functional performance associated with environmental change, especially if interactions and variance are altered. We can and should do better by keeping it complicated.

## Methods

### Data Sources and Test of Mechanism of Impact on Ecosystem Function

The manuscript contains unpublished data on the hydraulic activities of three ecosystem engineers. As detailed below these data on types of behaviors, durations, and impacts on porewater flux are derived from records of pressure sensors implanted into the sediment of containers with known numbers and sizes of animals of given species in the laboratory. The pressure sensor records were supplemented with behavioral data taken in the laboratory and in the field and from the literature (summary of data and sources: Supplementary Table S3). The behavioral data and porewater pressure data combined with knowledge of depth and rate of burrowing in the field and position within the sediment of each species allowed calculation of expected ammonium efflux for habitats with known densities of organisms. The expected values could then be compared to field sites where such values had been measured. Since each of the three species was predicted to affect ammonium efflux by very different mechanisms, this allowed a test of the proposed mechanism of impact on ecosystem function. One of the strengths of such a test is that rates derived in controlled laboratory conditions are being compared to rates measured in the field where there are potentially many sources of variation. Agreement between these very different sources of data is a robust test of the predictions and very important since it is mechanisms operating in the field that we ultimately wish to understand.

### Behaviors

Behaviors of *Austrovenus*, *Macomona* and *Echinocardium* were quantified with a combination of measurements of porewater pressure dynamics^23^,^26^,^37^, time lapse photography of the sediment surface^23^ and oxygen distributions inside the sediments measured with a time-resolved, luminescence lifetime imaging system^25^ (Table 1; Supplementary Table S3).

To identify the porewater pressure dynamics associated with particular behaviors of each infaunal species^23^,^26^,^37^, individuals were established in fresh native sediment with running seawater in the laboratory. Temperature was 17–19 °C with ambient light and salinity of 31–34. Tanks were either cores filled with native sediment containing one individual or larger tubs containing multiple individuals or antfarms with a transparent wall equipped with an oxygen optode. Aquarium size varied with size and activity of species; in all cases the width of the aquarium was sufficient to allow individuals to rotate easily without encountering an aquarium wall. Each tank contained a differential pressure sensor with the plenum open at the sediment water interface and the sediment sensor at approximately 5 cm within the sediment, and each tank was photographed at 15 or 30 s intervals by cameras focused on the sediment surface and in the case of the optode tanks the sediment of the tank side-wall as well as the optode film tank side-wall. Porewater pressure signals were recorded using an autonomous data logger (Persistor Instruments, Bourne MA)^16^,^18^. Clocks in the cameras and data loggers were synchronized to allow pressure signals to be related to observed changes in sediment topography and animal movement. Imagery was searched for time periods in which distinct behaviors were observed. Using ten such periods each from three different individuals, pressure-sensor records were scanned to determine whether the pressure waveforms associated with each behavior were identifiable^19^. Known waveforms for each behavior were then used to analyze the pressure records.

Individual *Macomona* and *Austrovenus* were established as single individuals in sediment cores (11.1 cm diameter, 10.5 cm deep, n = 16). Individuals were collected in the field and typically introduced into fresh field-collected sediment from which large shell debris and large organisms had been removed within 24 h of collection. The recording period was ~7 days^26^. Additional recordings of each species were made with single individuals in antfarm aquaria equipped with an oxygen optode (20 cm × 4.5 cm surface, 20 cm deep, optode 20 × 20 cm). Additional recordings of multiple *Austrovenus* were made in 34 × 25 × 20 cm deep containers equipped with a 20 × 13 cm optode. No differences in recordings were seen as a function of chamber type or number of animals present unless two individuals were simultaneously recorded or when the width of the chamber was too small to allow the individual to rotate. If animals appeared inhibited in their movements, the data were discarded.

*Echinocardium* is too large and mobile to be confined in 11.1 cm diameter cores and too globular in shape for effective use with antfarms. Ten to fifteen individuals were placed in two 34 × 25 × 20 cm deep containers fitted with one transparent wall with a 20 × 13 cm oxygen optode. One or two pressure sensors were placed within each tank. This procedure was repeated several times over several years.

#### Oxygen imaging

Oxygen distributions inside the sediments were measured by planar optode imaging using a luminescence lifetime imaging system^25^. Images were acquired in 15 s intervals.

#### Time lapse photography

Images of the tanks were taken with digital SLR cameras (Nikon D200 and D300) triggered by either autonomous time-lapse controllers (Digi-Snap, Harbortronics) or by the optode camera controller with Nikon images interspersed with the optode images to prevent illumination interference.

#### Porewater pressure measurements

The differential pressure sensors (Honeywell 26PC) are piezoresistive bridges that provide a voltage proportional to the pressure difference between the two sides of the sensor. Data were collected at 200 Hz. The pressure measurement side of the sensor was in direct contact with the sediment porewater, and the reference side of the sensor measured the ambient (hydrostatic) pressure within a water filled space within a PVC channel (plenum) that was in direct contact with the overlying water and isolated from the porewater^26^. Sensors were calibrated by varying the water heights on both sides of the sensor i.e. the plenum and the sediment side. The linear calibrations of the gauge pressure and the recorded voltage had R^2^ > 0.95.

### Sediment, chlorophyll *a*, oxygen, ammonium flux

#### Oxygen and ammonium efflux data

Incubations to determine sediment nutrient efflux were all done in the field. The *in situ* estimates of ammonium efflux and primary production were obtained for intact communities dominated by either *Austrovenus*, *Macomona* or *Echinocardium* from published studies conducted by co-authors (Supplementary Table S3) using similar methods^35^,^46^,^52^,^53^. The studies were selected to standardize for season (summer) and minimize variations in water temperature (incubation temperatures: *Austrovenus* 22 °C^35^, *Macomona* 23 °C^53^, *Echinocardium* 21 °C^46^,^52^). The site properties and latitude and longitude values are given in Supplementary Table S4. The ammonium efflux values obtained in these studies are in Table 2. In each study, light and dark benthic chambers were deployed in plots incubating the sediment and overlying water column for a 3–4 h period around a midday high tide on a calm and sunny day. Chamber sizes differed among the studies: *Austrovenus* and *Echinocardium:* 0.25 m^2^ surface area and enclosed 25 L overlying seawater; *Macomona:* 0.016 m^2^ and enclosed 0.85 L seawater. In all cases flux measurements were made over a 3 h period. Chamber deployment for the intertidal areas (*Austrovenus* and *Macomona* sites) began when incoming tidal waters were ~0.5 m deep so that the chambers could be deployed without trapped air bubbles. The incubation period was chosen to ensure O_2_ concentrations in dark chambers never dropped below 50% of the initial value, potentially inducing stress. Light chamber oxygen fluxes provide an estimate of net primary production (NPP (μmol O_2_ m^−2^ h^−1^), whereas, dark chambers block photosynthesis providing an estimate of total community metabolism and nutrient efflux in the absence of photosynthesis. The sum of the light and dark chamber O_2_ fluxes represents gross primary production (GPP). NPP and GPP were normalised by microphytobenthic biomass (sediment chl *a* content) to compare photosynthetic efficiency among sites. Ammonium and oxygen fluxes were determined from changes in solute concentrations determined in water samples collected from chambers during the incubation (N = 2–5). O_2_ concentrations were determined immediately using a calibrated handheld sensor, and ammonium samples were filtered, placed in the dark, and frozen until analysis on an auto-analyser (La Chat or Thermo Scientific Aquakem 200) following standard techniques^35^,^46^,^52^,^53^.

#### Sediment Data

Nutrient efflux, primary production and associated sediment properties (grain size, organic content, surficial sediment chlorophyll *a* concentration) were derived from field experiments: *Austrovenus*^35^; *Echinocardium*^46^,^52^. The data for *Macomona* are from Manukau Harbor and were part of a much larger study^41^,^53^. As noted in Supplementary Table S3, all the data are for summer or early fall conditions; winter data were excluded. Given the known negative effects of mud content on primary production^54^,^55^, sites with >20% mud content were also excluded. Sediment was sampled in each chamber with 2.5 cm ID, 2 cm deep cores to determine: organic content (percentage loss on ignition of dried sediments at 550 °C for 5 h), and grain size either following standard gravimetrical or laser diffraction (Multisizer) techniques. Methods for determining chlorophyll *a* content (per g of sediment dry weight) differed somewhat across the sources (determination spectrophotometrically after extraction in ethanol or fluorometrically following acetone extraction) but the methods give similar results^53^. In all cases when NPP or GPP were normalized by chlorophyll *a*, the value for the experimental site, not the site average for chlorophyll *a*, was used.

### Densities

*Echinocardium* and *Austrovenus* densities were determined in each 0.25 m^2^ chamber after incubation in which ammonium and oxygen efflux was measured; for *Macomona* the density was assessed from a 0.25 m^−2^ area that included the area covered by the smaller chambers. Densities are scaled up to per meter square and are presented in Table 2.

### Statistics

All analyses were done using several packages in R. Data were tested for normality (Shapiro-Wilks test), for homogeneity of variances (Bartlett, Levene, and Fligner tests), and examined graphically for patterned residuals. Deviations were corrected by transformation or by using a non-parametric test if transformation failed to correct the problem, see descriptions for each dataset in the Results as to whether a transformation was done.

## Additional Information

**How to cite this article**: Woodin, S. A. *et al*. Same pattern, different mechanism: Locking onto the role of key species in seafloor ecosystem process. *Sci. Rep*. **6**, 26678; doi: 10.1038/srep26678 (2016).

## Supplementary Material

Supplementary Information

## Figures and Tables

**Table 1:** Means of common bioadvective behaviors which affect properties beyond the volume immediately surrounding the body of the individual and the diffusional layer, derived from pressure sensor data.

Species	Behavior	Duration (min)	Frequency (h^−1^)	Integral of time pressurized (h^−1^)
*Macomona*	feeding/respiration	7.4	4.0	0.5
	burrowing	2.5	0.25	0.01
	defecation/pseudofecal expulsion	<0.1	4.6	<0.005
	siphon movement	3.9	0.6	0.04
*Austrovenus*	burrowing	uncommon except for initial burrowing into the sediment	rare	
	defecation/pseudofecal expulsion	<0.1	<1	
	pedal gape pulses (labial palp cleansing)	<0.1	9	<0.005
*Echinocardium*	none			0

**Table 2:** Ammonium excretion expected for the population versus measured ammonium efflux.

Species	Site	Density (ind m^−2^) (mean)	NH_4_^+^excretion	NH_4_^+^efflux in light (mean)	NH_4_^+^efflux in dark (mean)	NH_4_^+^efflux in dark – NH_4_^+^efflux in light
*Austrovenus*	Tuapiro Point, Tauranga	2246.2	179.7	74.6	105.8	31.2
*Macomona*	Manukau	224	10.3	87.0	165.5	78.5
*Echinocardium*	Martins Bay	10.3	0.37	13.3	2.3	−11
	MahuMoor, Mahurangi	16.8	0.60	26.8	21.4	−5.4
	Big Bay	23.25	0.84	11	6.5	−4.5
	mean	16.8	0.6	17.0	10.1	−7.0

Ammonium excretion from Lohrer (pers. comm) in μmol h^−1^ ind^−1^: 0.08 (*Austrovenus*) and 0.046 (*Macomona*). Ammonium excretion in *Echinocardium* assumed to be crudely 10% of oxygen consumption (0.356 μmol h^−1^ ind^−1^ for a standard 3 g DW individual^39^) so 0.036 μmol h^−1^ ind^−1^. See Supplementary Table S4 for latitude, longitude of sites. Data sources and time of year: *Austrovenus* Feb-March (22 °C, summer-early fall)^35^; *Macomona* January (23 °C, summer)^53^; *Echinocardium* Dec–Jan^46^ & March^52^ (21 °C, summer-early fall). Units of NH_4_^+^ are μmol m^−2^ h^−1^.

**Table 3:** Ammonium efflux expected for the population based on bioadvection or sediment turnover.

Species	Site	Density (ind m^−2^)	Advective bioirrigation	Sediment turnover by population (cm^3^ m^−2^ d^−1^)	Proportion reworked down to 5 cm (m^−2^ d^−1^)	Expected NH_4_^+^ efflux due to population bioadvection or sediment turnover (μmol m^−2^ h^−1^)
*Austrovenus*	Tuapiro Point, Tauranga	2246.2	negligible	negligible	na	na
*Macomona*	Manukau	224	12.9 mL h^−1^ ind^−1^ 2.9 L m^−2^ h^−1^ (by pop)	negligible	na	289
*Echinocardium*	Martins Bay	10.3	negligible	3820	0.08	6
	MahuMoorMahurangi	16.8	negligible	6039	0.12	10
	Big Bay	23.25	negligible	8241	0.16	14
	mean	16.8		6033	0.12	10

Population level sediment turnover rates from Fig. 5 in ref. 31. See Supplementary Table S4 for latitude, longitude of sites.
