# Supplementary material for: Same pattern, different mechanism: Locking onto the role of key species in seafloor ecosystem process
Source: Sci Rep. 2016 May 27;6:26678. doi: 10.1038/srep26678 (PMC4882525; doi:10.1038/srep26678)
Supplement: Supplementary Information [file srep26678-s1.docx]

**Supplementary Material for :**

**Same pattern, different mechanism: Locking onto the role of key species in seafloor ecosystem process**

Sarah A. Woodin^1*^, Nils Volkenborn^1,2^, Conrad A. Pilditch^3^, Andrew M. Lohrer^4^, David S. Wethey^1^, Judi E. Hewitt^4^, Simon F. Thrush^4,5^

^1^Department of Biological Sciences, University of South Carolina, 701 Sumter Street, Columbia, South Carolina 29208, USA

^2^School of Marine and Atmospheric Sciences, Stony Brook University, Stony Brook, New York 11794-5000, USA

^3^School of Science, Faculty of Science and Engineering, University of Waikato, Hamilton 3240, New Zealand

^4^National Institute of Water and Atmospheric Research, PO Box 11-115, Hamilton 3251, New Zealand

^5^Institute of Marine Science, University of Auckland, Private Bag 92091, Auckland, 1142, New Zealand

*email**:** [woodin@biol.sc.edu](mailto:woodin@biol.sc.edu)

**Supplementary Material**

**Background on animals**

The sedimentary habitats of New Zealand offer an interesting opportunity to compare impacts of ecosystem engineers: a surface-feeding, bulldozing disturbance agent, the urchin *Echinocardium cordatum*; a surface-feeding species which injects water into sediments at depth, driving bioadvection, the tellinid bivalve *Macomona liliana*; and a large suspension-feeder that forms dense groupings in the upper sediment layer, the foundation species the venerid bivalve *Austrovenus stutchburyi.* All three dominate large areas of muddy sands in terms of activities or biomass and are well-studied in terms of effects on fluxes, rates of movement, densities, and behaviors^1-13^. These intertidal (*Macomona* and *Austrovenus*) and shallow (c 5m) subtidal (*Echinocardium*) habitats thus provide an opportunity to explore impacts of very different biogenic drivers on ecosystem function. We use ammonium efflux as an indicator of seafloor N cycling and microphytobenthic productivity. All three species meet the criteria of being very important members of the assemblage where large reductions in density, biomass, or activity would alter ecosystem rates^14-15^. Fundamental differences in the bioturbation and feeding activities of the three large macrofauna are summarized in Supplementary Table S1 with predictions relative to ecosystem characteristics in Supplementary Table S2. Data sources and timing of data collection are summarized by species in Supplementary Table S3.

**Habitats**

The habitats in which the three species were abundant differed in several parameters. First, *Echinocardium* is primarily a subtidal animal although it occurs and can be dense in the low intertidal, while the other two species are commonly dense in the intertidal. Second, the organic contents of the sediments in the *Austrovenus* and *Echinocardium* beds were not significantly different from one another, while that of *Macomona* was significantly lower than either (ANOVA: F_2,9_ = 42.18, p < 0.000005; Tukey a posteriori tests: *Austrovenus:Echinocardium*  p > 0.15, *Austrovenus:Macomona*  p < 0.00005, *Echinocardium:Macomona* p < 0.0005) (Supplementary Table S4). The three types of seabed included here differed in mud content with that of *Macomona* significantly lower than those of *Austrovenus* or *Echinocardium* (Kruskal-Wallis rank sum test chi-squared = 8.48, df = 2, p < 0.01; Holm-Šidák test: *Austrovenus:Echinocardium*  p > 0.35, *Austrovenus:Macomona*  p < 0.05, *Echinocardium:Macomona* p < 0.025) (Supplementary Table S4). Note that sites with 20% or greater mud content were not included because primary productivity is reduced in sites with higher mud content^7,11-12^.

**References**

1. Hewitt, J. E., Thrush, S. F., Cummings, V. J., Pridmore, R. D. Matching patterns with processes: predicting the effect of size and mobility on the spatial distributions of the bivalves *Macomona liliana* and *Austrovenus stutchburyi*. *Mar. Ecol.- Prog. Ser.* **135,** 57-67 (1996).

2. Mouritsen, K. N. Intertidal facilitation and indirect effects: causes and consequences of crawling in the New Zealand cockle. *Mar. Ecol.-Prog. Ser.* **271,** 207-220 (2004).

3. Lohrer, A. M., Thrush, S. F., Gibbs, M.M. Bioturbators enhance ecosystem function through complex biogeochemical interactions. *Nature* **431,** 1092-1095 (2004).

4. Lohrer, A. M., Thrush, S. F., Hunt, L., Hancock, N., Lundquist, C. Rapid reworking of subtidal sediments by burrowing spatangoid urchins. *J. Exp. Mar. Biol. Ecol.* **321,** 155-169 (2005).

5. Lohrer, A. M., Halliday, N. J., Thrush, S. F., Hewitt, J. E., Rodil, I. F. Ecosystem functioning in a disturbance-recovery context: Contribution of macrofauna to primary production and nutrient release on intertidal sandflats. *J. Exp. Mar. Biol. Ecol.* **390,** 6-13 (2010).

6. Lohrer, A., Thrush, S., Hewitt, J., Kraan, C. The up-scaling of ecosystem functions in a heterogeneous world. *Sci. Rep.* **5,** 10349; DOI 10.1038/srep10349 (2015).

7. Sandwell, D. R., Pilditch, C. A., Lohrer, A. M. Density dependent effects of an infaunal suspension-feeding bivalve (*Austrovenus stutchburyi*) on sandflat nutrient fluxes and microphytobenthic productivity. *J. Exp. Mar. Biol. Ecol.* **373,** 16-25 (2009).

8. Jones, H. F. E., Pilditch, C. A., Bruesewitz, D. A., Lohrer, A. M. Sedimentary environment influences the effect of an infaunal suspension feeding bivalve on estuarine ecosystem function. *PloS ONE* **6,** e27065, 27061-27015 (2011a).

9. Jones, H. F. E., Pilditch, C. A., Bryan, K. R., Hamilton, D. P. Effects of infaunal bivalve density and flow speed on clearance rates and near-bed hydrodynamics. *J. Exp. Mar. Biol. Ecol.* **401,** 20-28 (2011b).

10. Volkenborn, N. *et al.* Intermittent bioirrigation and oxygen dynamics in permeable sediments: an experimental and modeling study of three tellinid bivalves. *J. Mar. Res.* **70,** 794-823 (2012).

11. Pratt, D. R., Lohrer, A. M., Pilditch, C. A., Thrush, S. F. Changes in ecosystem function across sedimentary gradients in estuaries. *Ecosystems* **17,** 182-194 (2014).

12. Pratt, D. R., Pilditch, C. A., Lohrer, A. M., Thrush, S. F. The effects of short-term increases in turbidity on sandflat microphytobenthic productivity and nutrient fluxes. *J. Sea Res.* **92,** 170-177 (2014).

13. Thrush, S.F. *et al.* Experimenting with ecosystem interaction networks in search of threshold potentials in real world marine ecosystems. *Ecology* **95,** 1451-1457 (2014).

14. Norkko, A., [Villnäs](http://www.nature.com/srep/2013/130912/srep02646/full/srep02646.html#auth-2), A., Norkko, J., Valanko, S., Pilditch, C. Size matters: implications of the loss of large individuals for ecosystem function. *Sci. Rep.* **3,** 2646-2652 (2013).

15. Norling, K., Rosenberg, R., Hulth, S., Grémare, A., Bonsdorff, E. Importance of functional biodiversity and species-specific traits of benthic fauna for ecosystem functions in marine sediment. *Mar. Ecol.- Prog. Ser.* **332,** 11-23 (2007).

21. Lohrer, A. M. *et al.*  Biogenic habitat transitions influence facilitation in a marine soft-sediment ecosystem. *Ecology* **94,** 136-145 (2013).

22. Pratt, D.R.  *et al.*  Detecting subtle shifts in ecosystem functioning in a dynamic estuarine environment.  *PLoS ONE* **10,** e0133914. doi:10.1371/journal.pone.0133914 (2015).

23. Huettel, M., Ziegis, W., Forster, S. Flow-induced uptake of particulate matter in permeable sediments. *Limnol. Oceanogr.* **41,** 309-322 (1996).

24. Santos, I. R., Eyre, B. D., Huettel, M. The driving forces of porewater and groundwater flow in permeable coastal sediments: A review. *Estuar. Coast. Shelf S.* **98,** 1-15 (2012).

25. Vopel, K., Vopel, A., Thistle, D., Hancock, N. Effects of spatangoid heart urchins on O_2_ supply into coastal sediment. *Mar. Ecol.-Prog. Ser.* **333,** 161-171 (2007).

26. Whitlatch, R. B., Hines, A. H., Thrush, S. F., Hewitt, J. E., Cummings, V. Benthic faunal responses to variations in patch density and patch size of a suspension-feeding bivalve. *J. Exp. Mar. Biol. Ecol*. **216,** 171-189 (1997).

27. Buchanan, J. The biology of *Echinocardium cordatum* from different habitats. *J. Mar. Biol. Assoc. U.K.* **46,** 97-114 (1966).

28. Lohrer, A. M., Chiaroni, L. D.,Thrush, S. F., Hewitt, J. E. Isolated and interactive effects of two key species on ecosystem function and trophic linkages in New Zealand soft-sediment habitats. New Zealand Aquatic Environment and Biodiversity Report No. 44. 69 pp. (2010).

Supplementary Table S1. Ecosystem engineering activities of the three species, living in muddy sands where physical advective forces occur only in the upper few centimeters of sediment if at all^23-24^.

| Species | Bioadvection | Bulldozing of upper sediment layers | Fresh organic material into sediment at depth | Surface deposit feeding | Suspension feeding |
| --- | --- | --- | --- | --- | --- |
| *Macomona*  *liliana* | Exhalent siphon at depth, flushes upper 5 to 10 cm, dependent on permeability of sediment ^10, this paper^. | Minimal | Defecates at depth within sediment, injecting organic material. | Feeding on surface with inhalant siphon up to 10 cm from body. | Occasional, flow dependent |
| *Austrovenus*  *stuchburyi* | Minimal ^this paper^ | Moves body up and down, lateral crawling reduced at high densities^26^. | No, feces and pseudofeces enhance the organic content of surficial sediments. | No | Obligate,  feeding activities may increase bottom shear stress ^8^. |
| *Echinocardium*  *cordatum* | Actively irrigate area around test with surface water, no indication of hydraulic forcing of sediment distal to body ^25, this paper^. | Actively bulldozes through upper sediment layers causing increase in surface roughness ^3-4^. | Buries surface material to the depth of the bulldozed trough. | Yes, with tube feet^27^ | No |

Supplementary Table S2. Summary of predicted changes in characteristics of ecosystems relating to productivity as a function of the occurrence of large infaunal ecosystem engineers. These species can affect productivity by the following mechanisms: 1. drive particle reworking in that they move sediment particles (*Echinocardium*), 2. are bioadvective species (*Macomona*), or 3. are dense foundation species not known to have either such activity (*Austrovenus*). Equivalent light regimes are assumed for the productivity comparisons. ‘M’ *Macomona*, ‘E’ *Echinocardium,* ‘A’ *Austrovenus.*

|  | Summary Predictions | Bioadvection (e.g. *Macomona*) | Particle movement (e.g. *Echinocardium*) | Neither activity (e.g. *Austrovenus*) |
| --- | --- | --- | --- | --- |
| Ammonium efflux in the dark | density based excretion:  A > M >> E  activities:  M >> E >> A | Excretion will not account for majority of ammonium efflux.  Water pumped into sediment at depth, expressing porewater. Ammonium efflux related to activity and density.  Expect greater measured efflux in dark when minimal microphytobenthos uptake. | Excretion will not account for majority of ammonium efflux.  Ammonium release from porewater is a function of depth of burrowing and type of burrowing and density; axial burrower so do not expect large release of porewater nutrients due to sediment reworking itself. Rugose surface is expected to increase diffusive efflux and physical advection and photosynthesis.  Expect increase in measured efflux in dark when minimal microphytobenthos uptake. | Excretion—very dense populations, excretion may account for majority of ammonium efflux.  Expect increase in measured efflux in dark when minimal microphytobenthos uptake. |
| Surface rugosity | E >> A > or ~ M | No change | Increase in surface area due to creation of troughs and mounds during burrowing^4,25^. Potential for increase in physical advection and diffusion and benthic photosynthesis. | At high densities, partial emergence plus feeding may increase apparent roughness of the bed^8^. |
| Standing crop of chlorophyll a | A > M > E | *Macomona* is a surface deposit feeder which may reduce standing stocks of microphytobenthos (chlorophyll a content of sediment). | *Echinocardium* is a surface deposit feeder which may reduce standing stocks of microphytobenthos, plus its burrowing may reduce standing stocks. | *Austrovenus* is a suspension feeder, does eat resuspended microphytobenthos. |
| Provision of organic matter within sediment | M >> E > A | *Macomona* defecates at depth within the sediment providing fresh organic material at 5 to 10 cm depth. | *Echinocardium* moves upper sediment layers laterally, reshaping the surface, but turnover rate and depth are minimal^4,25^. | *Austrovenus* may increase organic matter of surficial sediments through its feeding and defecation. |
| Benthic primary productivity | M > A >> E | Enhanced, fertilization by porewater expressed, function of the sediment permeability and density and size and activity of the individual, typically a volume with radius 3 to 5 body lengths. | Reduced in area of active feeding.  Enhanced due to provision of increased surface area (30% increase^25^) and some advection. | No enhancement from a sediment activity.  Enhancement from excretion of very large densities. |
| Benthic primary productivity scaled to chlorophyll a concentration | M ~ A ~ E  expect equivalent efficiency of PP |  |  |  |

Supplementary Table S3. Data sources by species and measurement type.

| Species | Bioadvection | Field Behaviors | State Variables and Ammonium Flux |
| --- | --- | --- | --- |
|  |  |  |  |
| *Macomona* | this paper, 10  (summer —January & February) | this paper | 22  (summer —January) |
|  |  |  |  |
| *Austrovenus* | this paper  (summer —January & February) | 8, 26 | 8  (summer and early fall— February & March) |
|  |  |  |  |
| *Echinocardium* | this paper  (summer —January & February),  25  (fall— March & April) | 3, 4, 27 | 5  (early fall—March),  6  (summer—December & January) |

Supplementary Table S4. State variables for the locales used on the North Island of New Zealand. In all cases these sites have high densities of the species listed. ‘Org. Content’ is organic matter content, measured as percent loss on ignition. ‘Mud Content’ is the percent by weight or volume of particles < 63 µm in size. For *Austrovenus* and *Macomona* ‘Mean’ is mean of site/time means. ‘Range’ is extremes of site/time means. Each site is represented by a minimum of 3 replicates. For *Echinocardium* ‘Mean’ is mean of sites where each site is represented by one sample. ‘Range’ is extremes of within site values except for *Echinocardium* where it is among sites. Time of year: *Austrovenus:* Feb-March (summer-early fall); *Macomona*: January (summer); *Echinocardium:* MahuMoor, Mahurangi: Oct-March (spring-early fall); Martins Bay and Big Bay: Dec-Jan (summer). If a mean is in bold followed by an asterisk, then the value was significantly different from the other two species (Tukey or Holm-Šidák post-hoc test p<0.05).

| Species | Estuary | Latitude, Longitude | Water  Depth  (m) | Org Content  (%) | Chl a  (μg g dw^-1^) | Mud Content  (%) |
| --- | --- | --- | --- | --- | --- | --- |
|  |  |  |  | Mean  [Range] | Mean  [Range] | Mean  [Range] |
|  |  |  |  |  |  |  |
| *Austrovenus* | Tuapiro Point, Tauranga^8^ | 37.49°S, 175.95°E | low intertidal | 3.2  [3.1-3.3] | **17.7***  [16.8-18.25] | 9.1  [7.8-10.2] |
|  |  |  |  |  |  |  |
| *Macomona* | Manukau^22^ | 36.93°S, 174.76°E | low to mid intertidal | **0.9***  [0.7-1.1] | **12.8***  [9.7-15.0] | **0.56***  [0.08-0.98] |
|  |  |  |  |  |  |  |
| *Echinocardium* | Martins Bay^6^ | 36.453°S, 174.770°E | shallow subtidal (6-8 m) | 2.2 | 6.4 | 13.2 |
|  | MahuMoor, Mahurangi^21,28^ | 36.509°S, 174.724°E | shallow subtidal (6-8 m) | 3.4 | 9.3 | 19.9 |
|  | Big Bay^6^ | 36.498°S, 174.743°E | shallow subtidal (6-8 m) | 2.1 | 6.6 | 4.5 |
|  | mean |  |  | 2.6 | **7.4***  [6.4-9.3] | 12.5  [4.5-19.9] |
